# *QuickStats:* Percentage* of Adults Aged ≥65 Years Who Received Care at Home From a Nurse or Other Health Care Professional During the Past 12 Months,^†^ by Age Group — National Health Interview Survey, United States, 2018

**DOI:** 10.15585/mmwr.mm6927a7

**Published:** 2020-07-10

**Figure:**
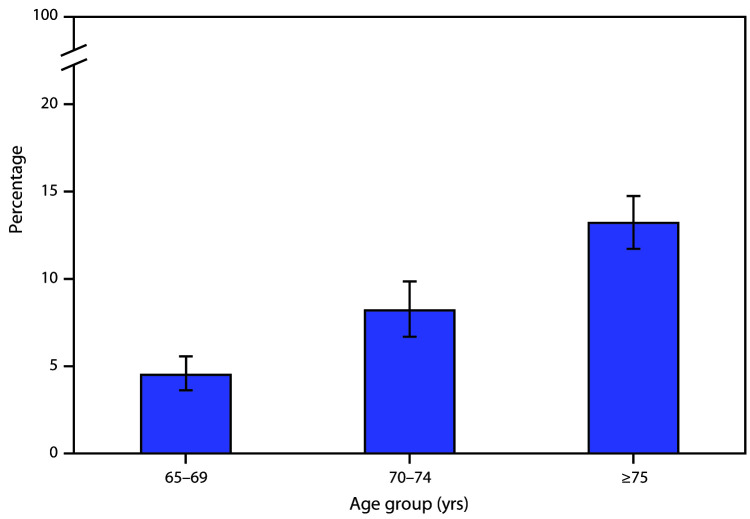
In 2018, the percentage of adults aged ≥65 years who received care at home from a nurse or other health care professional during the past 12 months increased with age from 4.5% for adults aged 65–69 years, to 8.2% for those aged 70–74 years and 13.2% for those aged ≥75 years.

**Source:** National Health Interview Survey, 2018 data. https://www.cdc.gov/nchs/nhis.htm.

